# Application of Sol–Gels Modified with Natural Plants Extracts as Stationary Phases in Open-Tubular Capillary Electrochromatography

**DOI:** 10.3390/gels8040198

**Published:** 2022-03-22

**Authors:** Jana Svobodová, Ivan Mikšík

**Affiliations:** Department of Translation Metabolism, Institute of Physiology of the Czech Academy of Sciences, 142 20 Prague, Czech Republic; ivan.miksik@fgu.cas.cz

**Keywords:** biologically important compounds, plant extracts, non-covalent interactions, sol–gel stationary phases, electrochromatography

## Abstract

Ethanol extracts of three widely growing plants were added to silica sol–gel solutions, which were subsequently applied as wall surface modifiers in inner quartz capillaries. Modified capillaries were used for open-tubular capillary electrochromatographic separation of nucleotides and amino groups containing biological compounds (neurotransmitters, amino acids and oligopeptides). The experiments were performed at physiological pH 7.40, and eventual changes of effective mobilities were calculated. Specific compounds characteristic for each plant were tested as sol–gel additives as well, and thus-modified capillaries were used for the separations of the same analytes under identical conditions. The aim of this study was to find out possible interactions between physiological compounds and extracts of freely available plants anchorded in the sol-gel stationary phase in the flowing system. Even though the amount of the modifier in each capillary is very small, basic statistical evaluation showed some not negligible changes in effective mobility of tested analytes. These changes were bigger than ±5% for separations of nucleotides in capillaries with curcuma, Moringa or the mixture of synthetic additives as the sol-gel aditive, and for separations of amino compounds where these changes varying by additive, analyte by analyte.

## 1. Introduction

Over the past few decades, numerous stationary phases have been widely used in open-tubular capillary electrochromatography (OT-CEC) separation [1]. Different types of natural or synthetic materials have been tested as the inner-surface capillary modifiers for the separation of a large spectrum of analytes [1,2]. Concurrently, another rapidly growing area of interest is the entrapment of biological materials into silica sol–gel matrices and the application of these matrices as biomaterials in bone tissue regeneration, drug delivery, biosensors, biomedical applications and many other applications [3,4,5,6,7,8,9,10]. The combination of the advantages of OT-CEC and the entrapping possibilities of the sol–gel technique (inner capillary wall modification) allows the possibility of tracking the interactions between incorporated natural substances and biological analytes in a relatively short time [11], which is the subject of our interest. Plant-derived products have been used for healing in many nations and cultures for centuries, and most current drugs originate from natural products [12]. Therefore, we examined the physiological importance and applications of three natural products—*Curcuma longa*, *Moringa oleifera* and *Hypericum perforatum*.

Turmeric, the dried rhizome of the plant *Curcuma longa*, is widely used in India and has extensive pharmacological activities, including neuroprotective, analgesic, anti-inflammatory, anticancer, antidiabetic, antithrombotic, antidiarrheal, antirheumatic, wound healing, antimicrobial, antiviral, antioxidant, antivenomous and many other activities [12,13,14,15,16]. The properties, chemical constituents, medicinal activities and potential benefits of biomedical applications of *Curcuma longa* have been comprehensively described [17]. Curcumin is the main bioactive compound in turmeric that confers the spice a healing effect [12,13]. Curcumin is a pleiotropic molecule providing multiple chemical interactions, such as extensive hydrogen and covalent bonding, metal chelation and π–π interactions [16]. Thanks to this property of its molecule, curcumin is able to interact with many molecular structures such as proteins, enzymes, lipids, DNA, RNA and transporter molecules [12,15]. Curcumin has also been shown to affect different types of ion channels and ion-channel-associated diseases [15,18].

The health benefits of Moringa (*Moringa oleifera*) as a representative of medicinal Indian herbs and natural nutrition, familiar in tropical and subtropical countries, have been published [19,20]. The content of its essential nutrients, health benefits, extraction methods, applications and utilization of Moringa as a superfood have been extensively described [19,20,21,22,23]. The activities of Moringa include antidiabetic, anti-inflammatory, antitumor, antihypertensive, antimicrobial and antioxidation activities and depend on the part of the plant used and its chemical composition [22]. The pharmacological action of Moringa with an emphasis on individual parts of the plant and their influence on human body systems from the perspective of traditional ayurvedic and Chinese medicine has been also extensively described [24]. Isoquercetin, one of the main active ingredients of *Moringa oleifera* leaf extract, has been shown to have antioxidant and anti-inflammatory effects [25,26].

St. John’s wort (*Hypericum perforatum*) is an herb that is widely distributed throughout the world. Its phytochemical constituents; pharmacological actions, such as antidepressant activity, antiparkinsonian, anticonvulsant, antidementia, antioxidant, anti-inflammatory, antimicrobial, anticancer and wound healing activities; and the toxicological effects (mechanism of action) of the active phytoconstituents have been reported [27]. *Hypericum perforatum* has been shown to be effective in treating various diseases of the nervous system [28,29]. The pharmacokinetic and pharmacodynamic properties of *Hypericum perforatum*, including plant–drug interactions during clinical measurements in various diseases, have been also described [30]. A detailed description of St. John’s wort, its historical background, its chemical composition and its antibacterial and antifungal activity have been summarized [31]. Deeper and more detailed insights into its chemistry, toxicology, pharmacology and physiological/clinical properties have also been reported [32,33,34]. Hypericin, one of the major components of St. John’s wort initially recommended for its antidepressant activity, was found to have better antiviral properties than neuroprotective properties and showed potential in antitumoral photodynamic therapy [35]. Nevertheless, hypericin can still be used for treating weak or moderate forms of depression and as a modulator of pain threshold due to its analgesic properties [36].

Considering the therapeutic uses of *Curcuma longa*, *Moringa oleifera* and *Hypericum perforatum*, we wanted to investigate the interactions between extracts of these three plants, and physiological compounds, i.e., nucleotides and substances containing amino groups. Plant extracts were added into the sol–gel procedure as a varying additive to prepare modified inner capillary walls. In addition to the three capillaries with the natural plant extracts, three other capillaries with the same basic sol–gel solution were prepared differing only in the additive, which was ethanol solution of the characteristic compound of the particular plant. The characteristic compounds of curcuma, St. John’s wort and Moringa are curcumin, hypericin and isoquercetin, respectively. We covered the inner walls of capillaries with the prepared sol–gels, conditioned under room temperature and investigated interactions between the physiological compounds using open-tubular capillary electrochromatography technique as the operational mode. The novelty of the presented article is in the combination of the sol–gel technology with the above-mentioned three plant extracts and their applications as stationary phases in the OT-CEC process, which to our best knowledge has not been published yet. A physiological buffer of pH 7.40 was applied and the eventual changes of effective mobility were calculated.

## 2. Results and Discussion

Several factors need to be considered at the beginning of this section.

First, we were observing interactions in flowing system. Secondly, electroseparations of these analytes are usually performed at lower (about 2.5) or higher (about 9.0) pH values of the running buffer. The electroosmotic flow is suppressed to minimum at low pH values, which provides a longer time for interaction, and increases with the increasing pH. Thus, the time for possible interaction shortens accordingly to the buffer pH set for analyses. Thirdly, the electroseparations of physiological compounds (substances) are seldom used in neutral buffer, because it is more difficult to separate them from each other, if an additive (i.e., sodium dodecyl sulphate) is not included in the buffer.

To approximately mimic physiological conditions, the separations were performed in 0.05 mol/L phosphate buffer at pH 7.40. Importantly, analysis of a group of analytes had to be carried out as fast as possible, ideally within one day—a requirement that was not always achieved because of the long duration of the analyses, particularly during nucleotide separations. Thus, migration times could shorten from day to day. This phenomenon could be caused by changes in the inner capillary surface due to chemical and/or structural modifications or wiping or leaking of an additive during separations, each of these phenomena differing by the number of repeated separations. However, unless otherwise stated, the separations of the final complete mixtures were performed at least three times in one day. The other reason for the shortening of the migration time, especially for the curcuma-modified capillary, is that curcumin is unstable at neutral and basic pH values and undergoes degradation to ferulic acid and feruloylmethane [12]. Most curcumin is rapidly degraded within 30 min when placed in phosphate buffer systems at pH 7.2 [12]. It is also worth mentioning that the process of sol–gel condensation could be accelerated by the use of a basic compound, e.g., aminopropyl triethoxysilane, but this was not applied to maintain the maximum access of the separated analytes to the components of the extract dispersed in a sol–gel layer.

**Figure 1:**
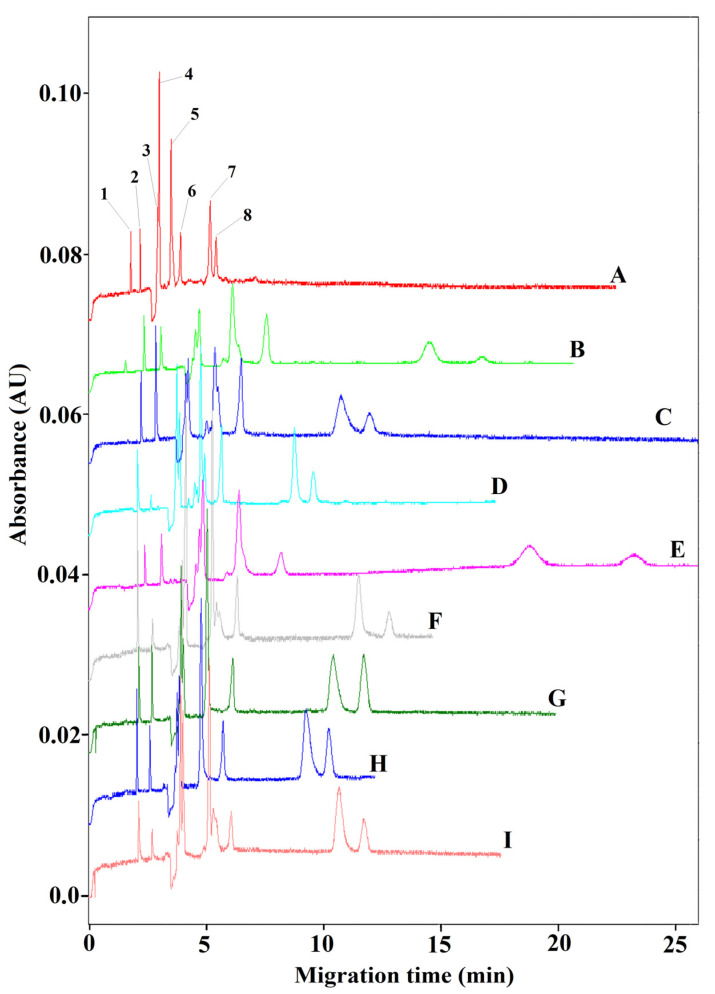
Separation of amino-group-containing compounds in the inner-surface-modified capillaries. Conditions: 0.05 mol/L Na_2_HPO_4_+1 mol/L HCl, pH 7.40, applied voltage 5 kV, 20 °C, normal polarity. (**1**) AChCl, (**2**) epinephrine, (**3**) L-Trp, (**4**) glutamine+A_1_, (**5**) L(En)+A_2_, (**6**) GDChA, (**7**) glutathione, (**8**) HIAA (A_1_ and A_2_ mean parts of angiotensin), injection time 3 s. (**A**) Sol–gel base, *n* = 3; (**B**) sol–gel + curcuma extract, *n* = 5; (**C**) sol–gel + Moringa extract, *n* = 3; (**D**) sol–gel + St. John’s wort extract, *n* = 7; (**E**) sol–gel + mixture 1 of extracts, *n* = 3; (**F**) sol–gel + curcumin, *n* = 3; (**G**) sol–gel + hypericin, *n* = 3; (**H**) sol–gel + isoquercetin, *n* = 4; (**I**) sol–gel + mixture 2 of specific compounds, *n* = 4; *n*, number of repeated analyses in a particular capillary.

The electrochromatograms obtained during the separations of amino group compounds are shown in Figure 1. Extreme changes in the separation of these substances were not observed, except for better resolution of glutathione (7) and HIAA (8), especially in capillaries modified with curcuma extract (line B, resolution R_7,8_ = 2.50) and a mixture of plant extracts (line E, resolution R_7,8_ = 3.62). The separation in the last-mentioned capillary modified with a sol–gel containing a mixture of plant extracts gave the longest migration time. The analytes migrated in the same order in all nine capillaries, which is why the only numerically described electrochromatogram is the first one on the top of Figure 1 (line A) of the pure sol–gel modifier. The resolution of glutathione (7) and HIAA (8) in all other capillaries varied by the interval R_7,8_ = 1.59–2.30.

**Figure 2:**
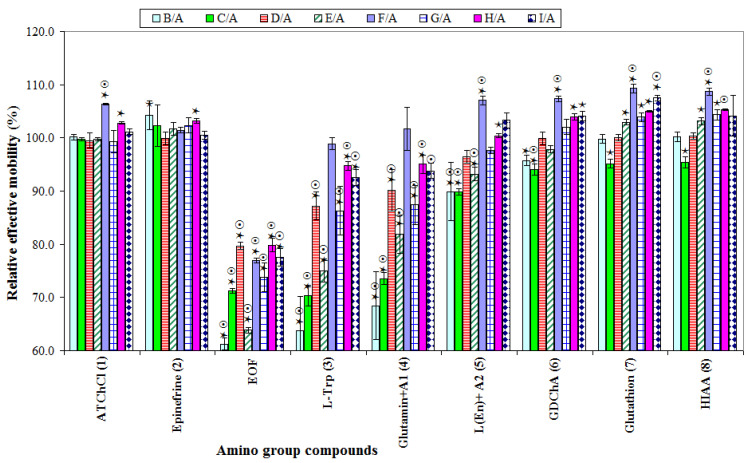
Relative effective mobility changes of amino group compounds in capillaries with modified sol–gels compared to 100% of the capillary with pure sol–gel without additive (line A). A *t*-test *p* ˂ 0.05 is marked by the symbol ★ above colored bars. The symbol ⊙ indicates that the values of the effective mobility changes were greater than ±5% of the standard deviation of Gaussian normal distribution, compared to the pure sol–gel capillary.

The percentage changes in the effective mobilities of amino group analytes compared to 100% mobility in a capillary modified with pure sol–gel are graphically presented in Figure 2. Interestingly, angiotensin showed two peaks in the electrochromatograms, regardless of the type of capillary installed for separation. Thus, the two peaks of angiotensin in Figure 1 and Figure 2 are marked as A_1_ and A_2_ in both the electrochromatogram and graphical representation, respectively. Figure 2 also shows that the two positively charged analytes, i.e., acetylcholin chloride (AChCl) (1) and epinephrine (2), migrate through the capillaries almost untouched by the inner-wall modifiers, in contrast with the negatively charged anions migrating behind electroosmotic flow (EOF), which seemed to be more affected by the sol–gel modifier.

**Figure 3:**
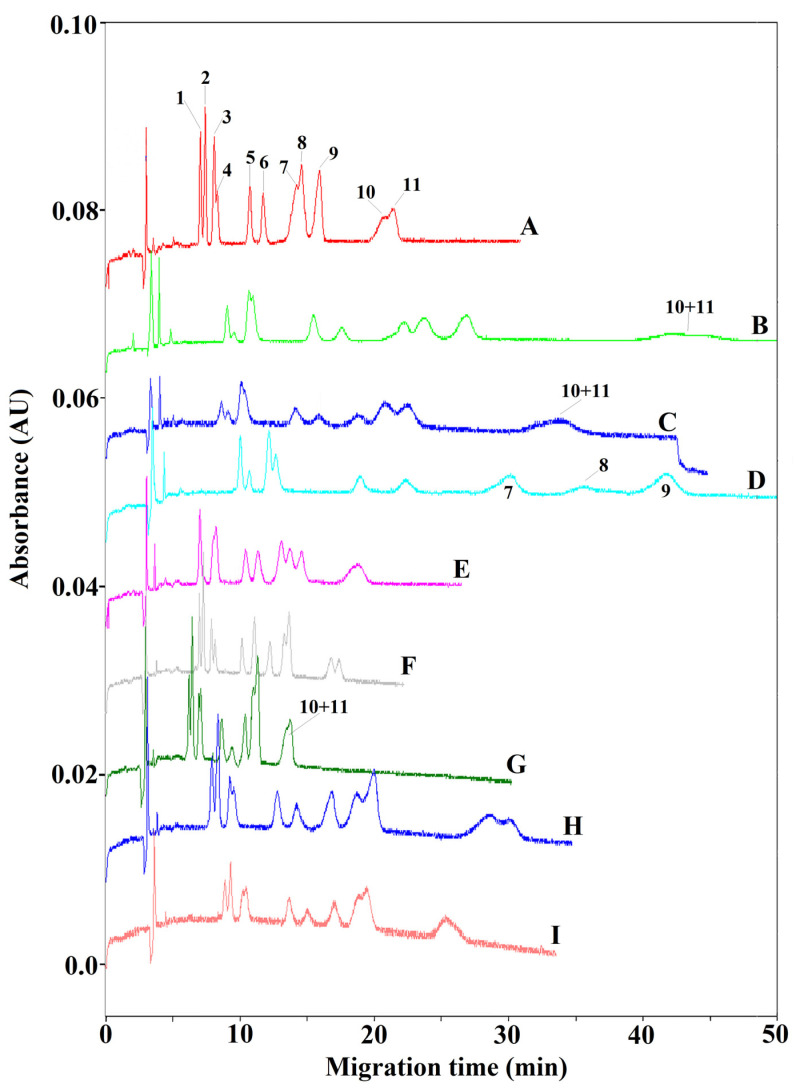
Separation of nucleotides in modified capillaries. Conditions: 5 kV, 20 °C, normal polarity, running buffer 0.05 mol/L Na_2_HPO_4_ + 1 mol/L HCl, pH 7.40, injection 3 s. (**1**) GMP, (**2**) AMP, (**3**) CMP, (**4**) UMP, (**5**) GDP, (**6**) ADP, (**7**) GTP, (**8**) CDP, (**9**) ATP, (**10**) CTP, (**11**) UTP. (**A**) Sol–gel base, *n* = 4; (**B**) sol–gel + curcuma extract, *n* = 3; (**C**) sol–gel + Moringa extract, *n* = 3; (**D**) sol–gel + St. John’s wort extract, *n* = 2; (**E**) sol–gel + mixture 1 of extracts, *n* = 5; (**F**) sol–gel + curcumin, *n* = 3; (**G**) sol–gel + hypericin, *n* = 4; (**H**) sol–gel + isoquercetin, *n* = 3; (**I**) sol–gel + mixture 2 of specific compounds, *n* = 4; *n*, number of repeated analyses in a particular capillary.

Similarly, the electropherograms of separations of nucleotides in modified capillaries are summarized in Figure 3. In capillary modified with sol–gel containing St. John’s wort extract, CTP and UTP did not elute from the capillary in the required time of 60 min (Line D). Furthermore, none of the modified capillaries contributed to better resolution; this was especially true of CTP (10) and UTP (11), which usually migrated in one peak or only partially divided.

**Figure 4:**
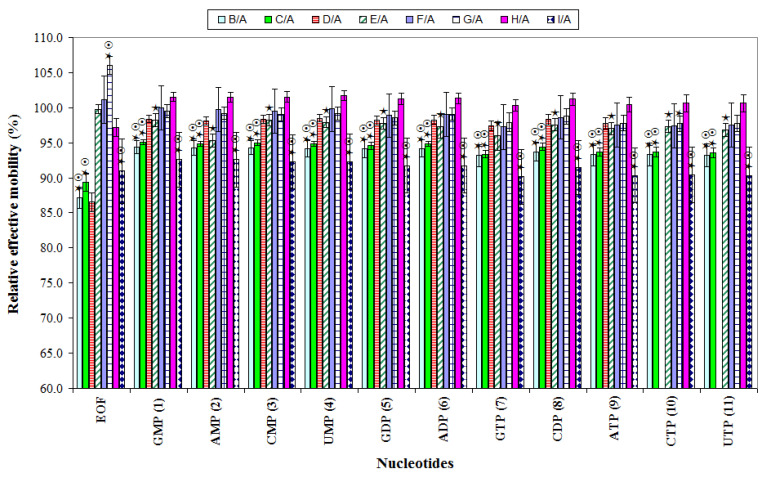
Relative effective mobility changes of nucleotides in capillaries with modified sol–gels compared to 100% of the capillary with pure sol–gel without additive (line A). A *t*-test *p* ˂ 0.05 is marked by the symbol ★ above the colored bars. The symbol ⊙ indicates that the values of the effective mobility changes were greater than ±5% of the standard deviation of the Gaussian normal distribution, compared to the pure sol–gel capillary.

The changes in effective mobilities are graphically represented in Figure 4, where the mobilities of nucleotides calculated in the pure sol–gel capillary are taken as 100% again. A *t*-test revealed that the most significant changes of effective mobility of individual compounds, marked with ★, were registered for capillaries modified with curcuma extract (line B), Moringa extract (line C) and a mixture of synthetic modifiers (line I). The same three capillaries showed changes greater than 5% (more than the statistical standard deviation) in the effective mobility of nucleotides (marked as ⊙) compared to the pure sol–gel capillary with no additives. As in the separation of amino group compounds, the order of nucleotides migrating to the detector remained the same, which is why the complete numerical description is only for the first capillary (line A) modified with pure sol–gel with no additive. Other specifically numbered electrochromatograms described nucleotides migrating broadly (line B and C) or undetectably (line D). The biggest differences in resolution were noted for substances GTP (7), CDP (8) and ATP (9), as follows: the resolution between GTP (7) and CDP (8) varied by the interval R_7,8_ = 1.26–2.3 in lines C, D, F, G, H and I, while CDP (8) and ATP (9) were best distinguished in lines A, B and D with R_8,9_ = 1.2–2.37. Specifically, the resolution R_7,8_ = 2.3 in lines D (St. John’s wort) and F (curcumin), and R_8,9_ = 1.93 in the capillary modified with curcuma extract (line B) and R_8,9_ = 2.37 in the capillary modified with St. John’s wort extract (line D).

Let’s summarize the overall positive results. It could be assumed that the effect of the plant extract anchored in the sol–gel was greater than the effect of the plant-dominant substance alone. Even though the amount of the dominant substance in an extract is much lower than when used as a single synthetic equivalent, and lowers again because of its dilution in the sol-gel solution and creating only thin layer on the wall, these trace amounts still seem to have not negligible effect on the separation. Notable changes of the effective mobility might be probably caused by the multiple interactions with the many extract components. Interactions between the modifier and an analyte took place, particularly for amino group-possessing compounds, but these varied from capillary to capillary, analyte to analyte. The sol-gel OT-CEC approach can be used to separate amino compounds at neutral conditions that are not possible with unmodified capillaries and can be tailored by various additives. In addition, we chose this method mainly as a way to monitor the additive–analyte interaction, not primarily to improve the separation.

The most significant changes in nucleotides separations were noted for capillaries modified with curcuma, Moringa and mixture of synthetic additives.

Several factors may play a role in all these changes mentioned above: the possible character and quantity of non-covalent interactions between the analyte and an additive (e.g., host-guest interaction, metal-ligand coordination, hydrogen bonds, π–π stacking), and the higher number of potential reactants (components) in a natural modifier. Another aspect, better or worse, of the separations in modified capillaries, is the peak broadening, whether a natural or synthetic additive was added to the sol–gel. It might be brought about by narrowing of the capillary inner surface due to the layer of the sol-gel and eventual interactions with the modifier.

The advantages of the stationary phase thus prepared for OT-CEC include easy preparation of the plant ethanol extract and the sol–gel solution, easy modification process and the fact, that the modified sol–gels do not interfere with PDA detection.

Of course, there are some undesirable facts. Firstly, the possibility of unstable modification caused by either leaking of the extract or the mechanical loss of the modifier.

Secondly, investigation of interactions of the test analytes with each of many substances present in the extract would be time consuming. In this article, we focused on the question/answer of whether the addition of the whole extract or a dominant compound causes any noticeable change at all or not.

Thirdly, a natural or synthetic additives fastened into the sol–gel modifier may lose a degree of freedom, thus losing their ability to form spatial interactions.

Because this methodology of monitoring possible interactions is rather unique, it is difficult to compare the obtained results with other similar publications.

## 3. Conclusions

The aim of this work was to determine the capability of biological compounds to interact with plant extracts using OT–CEC as an experimental method to approximately mimic interactions in biological systems. While some aspects of the study require improvement—e.g., more stable sol–gel modification, longer interaction times, higher concentration of either natural or synthetic additives etc.—partial interactions were demonstrated and calculated under near-physiological conditions. The interaction of the whole herb extract with the compounds of this model system can consist even in low doses of all active substances present in it together. Synergistic interactions between the components of individual herbs or mixtures of herbs are an important part of their therapeutic efficacy. And since the number of possible plant extracts of enormous number of plants all around the world is almost unlimited and offers a vast number of combinations, we can fine-tune the proportions of the basic sol–gel reaction components and various additives following the required purpose of its final applications.

## 4. Materials and Methods

### 4.1. Chemicals and Accessories

The nucleotides in the form of sodium salts were obtained from Sigma-Aldrich (Munich, Germany) except AMP from Merck (Darmstadt, Germany). Tetraethyl orthosilicate (TEOS) was obtained from Sigma-Aldrich as well as synthetic additives, i.e., curcumin, hypericin and isoquercetin (quercetin-3-glucoside), amino group-containing physiological compounds except for L-glutathione (Alexis Corp., Lausen, Switzerland) and glutamine (Fluka, Fluka Chemie AG, Sigma-Aldrich, Milano, Italy). Liquid chemicals, i.e., ethanol (EtOH) and chemicals for preparing the disodium hydrogen phosphate (Na_2_HPO_4_) buffer were obtained from Lach-Ner (Lach-Ner, Neratovice, Czech Republic), and hydrochloric acid (HCl) was obtained from Sigma-Aldrich. Parafilm (Bemis, WI, USA) and plastic syringes (Chirana, Stará Turá, Slovakia) were also often needed. Plastic materials such as pipette tips (Vertex, CA, USA), Eppendorf tubes (Eppendorf AG, Hamburg, Germany) and nylon syringe filters (13 mm, 0.22 µm, Labicom, Olomouc, Czech Republic) were used as well. Milli-Q water from a Millipore Direct-Q UV3 Water Purification System (Millipore, Bedford, MA, USA) was used for all experiments. The solid plant parts were obtained from a house garden (dried leaves of St. John’s wort), an internet shop (crushed *Moringa oleifera* leves, www.iswari.com, accessed on 9 February 2022) and a food shop (*Curcuma longa* in the form of crushed dried turmeric). The liquid coolant for capillary electrochromatographic analyses was Fluorinert™ FC-770 (3M™, Zwijndrecht, Belgium).

### 4.2. Electrochromatographic Analyses

Electrochromatographic analyses were performed on the Beckmann Coulter P/ACE 5500 (Fullerton, CA, USA) apparatus. Detection with a photodiode array (PDA) detector was tuned to the 254 nm wavelength for nucleotides and 214 nm for amino group-containing physiological compounds, with a bandwidth of 10 nm. Alternatively, two cartridges with slightly different widths of detection windows were used: 100 µm × 200 µm or 100 µm × 800 µm. Fused silica capillaries (Polymicro Technologies, Phoenix, AZ, USA) with a total length of 27 cm and an effective length of 20.5 cm (75 µm I.D. and 375 µm O.D.) were used for all separations. The injection was performed with an applied pressure of 3.4 kPa.

### 4.3. Preparation of Modified Capillaries for Open-Tubular Capillary Electrochromatography

#### 4.3.1. Preparation of the Sol–Gel Additives

**Scheme 1:**
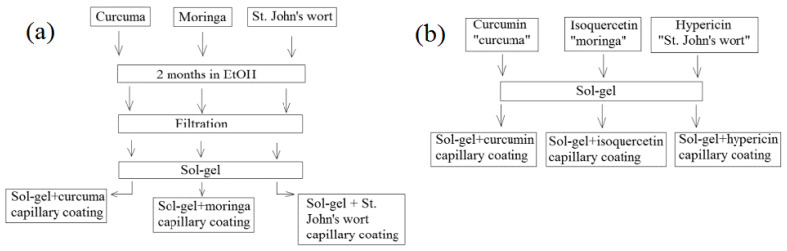
Experimetal design. (**a**): Sol-gel experiments with plant extracts, (**b**): Sol-gel experiments with synthetic additives.

The dried plant parts were immersed in pure ethanol (EtOH) to yield the following final concentrations: curcuma (5.257 mg/mL), St. John’s wort (67.666 mg/mL) and Moringa (109.2 mg/mL). The dried plants were left to leach in ethanol for at least 2 months at room temperature in ground glass beakers covered with aluminum foil to prevent light access. The liquid fraction (extract) was then aspirated with a syringe and filtered to a vial from which the appropriate amount was used for the sol–gel modification procedure. The filtered extracts of the natural plants were optically clear and had vibrant colors: bright yellow for curcuma, blood red for St. John’s wort and grass green for Moringa. The three synthetic additives, curcumin, isoquercetin and hypericin, were dissolved in ethanol to yield a concentration of 1 mg/mL. The mentioned process is simply illustrated in the Scheme 1.

#### 4.3.2. Modification of the Inner Capillary Surface

First, the fused silica capillary was prewashed in the following steps, each for 10 min: Milli-Q water, 1 mol/L NaOH, Milli-Q water, 1 mol/L HCl, Milli-Q water, ethanol, followed by an air flush. The standard basic solution of a sol–gel contained the following: 50 µL Milli-Q H_2_O, 100 µL EtOH, 50 µL TEOS, 50 µL of an additive and 20 µL 0.1 mol/L HCl. All ingredients were mixed, vortexed and left to react for 20 min. After a transparent clear solution was obtained, the capillary was washed with the solution using suction as follows: one end of the capillary was inserted into home-made tapering plastic tubing connected to an injection syringe, and the other end was immersed in an Eppendorf vial containing the sol–gel solution. After the solution was vacuum-aspirated at the syringe end for 30 min, both ends of the capillary were sealed with parafilm and left at room temperature overnight. To prepare a capillary modified with pure sol–gel without any additive, 50 µL of EtOH was added to the sol–gel solution instead of the additive. For the three either plant-based or synthetic additives (i.e., curcuma or curcumin, Moringa or isoquercetin and St. John’s wort or hypericin), 50 μL of each ethanol solution was added to the solution. The mixture of natural plant extract additives contained 16.6 μL of each extract (16.6 μL of curcuma extract, 16.6 μL of Moringa extract and 16.6 μL of St. John’s extract), giving final 50 μL of additive (mixture 1). Similarly, the mixture of synthetic plant-specific additives contained 16.6 μL of each characteristic compound dissolved in EtOH (1 mg/mL) to give 50 μL of additive again (mixture 2). On the next day, the rest of the unreacted inner-wall modifier was removed from the capillary with a syringe. The capillary was then flushed with air and nitrogen (5 min each, pressure 200 kPa) and left open at room temperature until experiments had been performed (usually 4 weeks).

#### 4.3.3. Conditioning (Stabilization) of Capillaries and Electrochromatographic Separation

After modification and rest at room temperature, the capillaries were prepared for the separation experiments. Each capillary was first washed with water for 5 min and then washed with the mobile phase (buffer) for 10 min. All experiments were performed in 0.05 mol/L disodium hydrogenphosphate solution at a pH of 7.40, adjusted with 1 mol/L hydrochloric acid.

The basic concentration of the samples was 0.1 mg/mL for compounds with amino groups and 0.5 mg/mL for nucleotides. The separations were performed in a step-by-step process: initially, 20 μL of pure distilled water was used in a microvial with 2.5 μL of the first analyte, and the electrochromatographic separation was left to run. Next, another 2.5 μL of the other analyte was added to the first analyte in a microvial, and the separation proceeded again. Thus, the mixture of analytes was separated sequentially to verify the migration time.

### 4.4. Analytes

Simple compounds important for physiological processes were chosen as the first test set.

The following compounds were dissolved in Milli-Q water to a concentration of 1 mg/mL: acetylcholine chloride (AChCl) (neurotransmitter), epinephrine (hormone and neurotransmitter), hydroxyindole acetic acid (HIAA) (serotonine metabolite), L-tryptophane (L-Trp) (essential amino acid), glutamine (non-essential amino acid), angiotensin fragments 1–7, Asp–Arg–Val–Tyr–Ile–His–Pro (blood pressure control component), Leu-enkephalin (L(En)), Tyr–Gly–Gly–Phe–Leu (endogenous neurotransmitter), glycodeoxycholic acid (GDChA) (bile acid) and glutathione (antioxidant). Because the absorbance at the concentration of 1 mg/mL was too high, the analyte stock solutions were diluted 10 times to a final experimental concentration of 0.1 mg/mL.

Nucleotides used were as follows: uridine 5′-monophosphate (UMP), adenosine 5′-monophosphate (AMP), guanosine 5′-monophosphate (GMP), cytidine 5′-monophosphate (CMP), adenosine 5′-diphosphate (ADP), guanosine 5′-diphosphate (GDP), cytidine 5′-diphosphate (CDP), uridine 5′-triphosphate (UTP), adenosine 5′-triphosphate (ATP), guanosine 5′-triphosphate (GTP) and cytidine 5′-triphosphate (CTP). The final concentration of nucleotides dissolved in water and used for experiments was 0.5 mg/mL.

### 4.5. Mathematical Calculations

The effective mobilities of individual analytes were calculated according to the equation

μ_eff_ = μ_app_ − μ_EOF_ (1)

(μ_eff_ is effective mobility, μ_app_ is apparent mobility and μ_EOF_ is mobility of electroosmotic flow), while μ = $\frac{l.L}{t.V}$ (cm^2^/min.V) (*l* is effective capillary length to the detector, *L* is total capillary length, *t* is migration time and *V* is applied voltage).

Resolution of two consecutive peaks was calculated according to the equation

(2) $$R_{1,2}=\frac{t_{2}-t_{1}}{0.5(w_{1}+w_{2})}$$

*t* is migration time of an analyte and *w* is baseline peak width. 

### 4.6. Statistics

*t*-test was set and calculated as programmed in the MS Excel, Function *t*-test, (i.e., field 1, field 2, 2 sides, type 3), the standard deviation was calculated in MS Excel program as well.

## Data Availability

Not applicable.

## Abbreviations

AChCl, acetylcholine chloride; ADP, adenosine 5′-diphosphate; AMP, adenosine 5′-monophosphate; ATP, adenosine 5′-triphosphate; CMP, cytidine 5′-monophosphate; CDP, cytidine 5′-diphosphate; CTP, cytidine 5′-triphosphate; GDChA, glycodeoxycholic acid; GDP, guanosine 5′-diphosphate; GMP, guanosine 5′-monophosphate; GTP, guanosine 5′-triphosphate; HIAA, hydroxyindole acetic acid; L(En), leucine-enkephalin; L-Trp, L-tryptophane; OT-CEC, open-tubular capillary electrochromatography; TEOS, tetraethyl orthosilicate; UMP, uridine 5′-monophosphate; UTP, uridine 5′-triphosphate.
